# Human dendritic cell targeting peptide can be targeted to porcine dendritic cells to improve antigen capture efficiency to stimulate stronger immune response

**DOI:** 10.3389/fimmu.2022.950597

**Published:** 2022-08-19

**Authors:** Tian Xia, Huizhu Yang, Yuyao Guo, Tiantian Guo, Lingxiang Xin, Yanping Jiang, Wen Cui, Han Zhou, Xinyuan Qiao, Xiaona Wang, Jiaxuan Li, Zhifu Shan, Lijie Tang, Li Wang, Yijing Li

**Affiliations:** ^1^ College of Veterinary Medicine, Northeast Agricultural University, Harbin, China; ^2^ Division of Bacterial Biologics Testing (I) China Institute of Veterinary Drug Control (IVDC), Beijing, China; ^3^ China Ministry of Agriculture Key Laboratory of Animal Pathogen Biology, Northeastern Science Inspection Station, Harbin, China

**Keywords:** porcine dendritic cells, human dendritic cells-targeting peptide, probiotic bacteria, porcine epidemic diarrhea virus, antigen-presenting

## Abstract

Dendritic cells (DCs) play a key role in the natural recognition of pathogens and subsequent activation of adaptive immune responses due to their potent antigen-presenting ability. Dendritic cell-targeting peptide (DCpep) is strongly targeted to DCs, which often express antigens, to enhance the efficacy of vaccines. Our previous study showed that recombinant *Lactobacillus* expressing human DCpep could significantly induce stronger immune responses than recombinant *Lactobacillus* without DCpep, but the mechanism remains unclear. In this study, the mechanism by which DCpep enhances the immune response against recombinant *Lactobacillus* was explored. Fluorescence-labeled human DCpep was synthesized to evaluate the binding ability of human DCpep to porcine monocyte-derived dendritic cells (Mo-DCs) and DCs of the small intestine. The effects of Mo-DC function induced by recombinant *Lactobacillus* expressing human DCpep fused with the porcine epidemic diarrhea virus (PEDV) core neutralizing epitope (COE) antigen were also investigated. The results showed that human DCpep bind to porcine DCs, but not to porcine small intestinal epithelial cells. Human DCpep can also improve the capture efficiency of recombinant *Lactobacillus* by Mo-DCs, promote the maturation of dendritic cells, secrete more cytokines, and enhance the ability of porcine DCs to activate T-cell proliferation. Taken together, these results promote advanced understanding of the mechanism by which DCpep enhances immune responses. We found that some DCpeps are conserved between humans and pigs, which provides a theoretical basis for the development of a DC-targeted vaccine.

## Introduction

Common porcine enteroviruses include porcine epidemic diarrhea virus (PEDV), transmissible gastroenteritis virus, porcine rotavirus, the newly discovered porcine delta corona virus, porcine kobuvirus, and the newly discovered swine acute diarrhea syndrome coronavirus. These enteroviruses can cause vomiting, diarrhea, and dehydration in infected piglets, resulting in many piglets dying and huge economic losses to the pig industry (1). The porcine intestinal mucosa is the main entry route for porcine enteroviruses to invade the organism. Therefore, the development of oral vaccines that can effectively induce mucosal immunity represents a crucial strategy for the prevention of enterovirus infection in piglets. (2).

The most common and important antigen-presenting cells involved in gastrointestinal immunity are dendritic cells (DCs) (3). The majority of DCs reside in the subepithelial dome (SED) region below the follicle-associated epithelium (FAE) and can take up antigens directly from M cells. Therefore, DCs act as the strongest antigen-presenting cells and when the virus enters the organism it is first recognized by DCs (4–6). Most DCs are in an immature state; immature DCs exhibit extremely strong antigen phagocytosis and differentiate into mature DCs upon uptake of antigens (including *in vitro* preparations) or stimulation by certain factors. If pattern recognition receptors (PRRs) detect pathogen-associated molecular patterns (PAMP) or damage-associated molecular patterns (DAMP), they activate DCs or develop maturation (7). Upon arrival at a draining lymph node, immature DCs can complete their development into mature DCs (7–9). DCs can modulate adaptive immunity (10). After maturation, they upregulate mechanisms of antigen presentation, including major histocompatibility complex (MHC)-II, costimulatory molecules and pro-inflammatory cytokines, which then migrate to the T-cell area of secondary lymphoid tissue where they stimulate antigen-specific T cells. (11, 12).

Targeting molecules that mediate antigen binding to DCs can improve the efficiency of DCs in recognizing and ingesting antigens, thus enhancing the immune response (13). Therefore, DC-targeted molecules are of great significance in the development of DC-targeted drugs and vaccines. At present, two principal strategies to explore DC-targeted molecules are: selection from known antibodies and ligands that interact with DC, such as CD154, CTLA4, heat shock protein (HSP), and DEC-205 (14–17), and the use of phage display technology to screen unknown DC-targeted molecules (18). A previous study described a vaccine strategy that utilized *Lactobacillus acidophilus* to deliver *Bacillus anthracis* protective antigen (PA) to human DCs *via* specific 12-mer DC peptides (DCpep) (19). The human-derived DCpep also recognized conserved regions of its ligand on birds, horses, dogs, and the cat family (13). Currently, a promising strategy is to utilize *Lactobacillus* to express DCpep conjugated with immunogenic antigens to elicit robust immune responses; however, the mechanism by which they promote immunity remains unclear (20–24).

In the present study, we used recombinant *Lactobacillus* pPG-COE-DCpep/*L393* expressing DCpep and the core neutralizing epitope (COE), a protective antigen of PEDV, as experimental models to explore the mechanism by which human DCpep enhances the immune effect of antigens on the capture efficiency, differentiation, and presentation of DCs. Our study may promote research on DC-targeting strategies and provide a new theoretical mechanism for the development of DC-targeted vaccines.

## Materials and methods

### Bacterial strains

The recombinant *Lactobacillus* strains (pPG/*L393*, pPG-COE /*L393*, pPG-COE-DCpep/*L393*) used in this study were prepared and constructed as previously described (25). The recombinant lactobacilli were cultured in Man, Rogosa, and Sharpe broth (MRS; Hopebol, Qingdao, China) at 37 °C for 15 h without shaking. Subsequently, while the recombinant *Lactobacillus* and cells were co-cultured, each *Lactobacillus* was inoculated in fresh MRS and incubated at 37 °C for 7 h until the mid-log phase. *Lactobacillus* precipitates were obtained by centrifugation, washed with Roswell Park Memorial Institute (RPMI) 1640 medium (Gibco, Grand Island, NY, USA), and added to monocyte-derived dendritic cells (Mo-DCs).

### Synthesis of peptides

Human DC-binding peptides (FYPSYHSTPQRP) and a control peptide (EPIHPETTFTNN) were synthesized using 9-fluorenylmethyoxycarbonyl chemistry and purified using high-pressure liquid chromatography to 95% purity by GenScript (Nanjing, Jiangsu) (26). The C-terminal was labeled with fluorescein isothiocyanate (FITC).

### Isolation and validation of Mo-DCs

Peripheral blood mononuclear cells (PBMC) were isolated from the blood of healthy pigs by Ficoll gradient centrifugation as previously described (27). PBMC (10^7^/well) were cultured in complete RPMI 1640 medium with 10% fetal calf serum (Gibco, Grand Island, NY, USA) in six-well plates at 37 °C for 6 h. Non-adherent cells were removed by multiple washes in RPMI 1640 medium and frozen for autologous mixed lymphocyte reaction (MLR) experiments. Adherent monocytes were washed thrice in RPMI 1640 medium and cultured with 20 ng/mL recombinant porcine granulocyte-macrophage colony stimulating factor (GM-CSF) and interleukin-4 (IL-4) (R&D Systems, Wiesbaden, Germany) in complete RPMI for 6 d. Maturation of immature (im)Mo-DCs was stimulated by supplementation with 2 μg/mL lipopolysaccharide (LPS, Sigma-Aldrich, St. Louis, MO, USA) for 24 h. Surface expression of the marker molecules CD172a and MHC-II (Abcam, Cambridge, UK) was analyzed by fluorescence microscopy (Bio-Rad, CA, USA) on 5th day. CD172a, MHC-II, and CD80/CD86 (Abcam, Cambridge, UK) expressed on the surface of immature (im) or mature (m)Mo-DCs were analyzed using flow cytometry.

### Determination of binding ability of human DCpep to porcine DCs with fluorescence microscopy and flow cytometry

#### Fluorescence microscopy

To evaluate the binding of human DCpep to porcine DCs, Mo-DCs and intestinal epithelial cells (IPEC), after discarding the culture medium, were washed thrice with PBS and incubated with FITC-conjugated human DCpep or control peptide for 30 min at 4 °C. After washing thrice with PBS, the cells were observed under a ZOE fluorescence microscope (Bio-Rad, Hercules, CA, USA).

To verify that human DCpep binds to small intestinal-DCs of piglets, frozen sections of piglet small intestine Fc receptors were blocked in 10% goat serum in PBS for 30 min at 37 °C. Anti-pig CD172a-PE and FITC-conjugated peptides were added to the frozen sections for 30 min at 37 °C. The cell nuclei were then stained with 4,6-diamidino-2-phenylindole (DAPI) solution (Invitrogen, USA) for 5 min at 37 °C and washed thrice for 5 min with fresh changes of TBS-Tween. The slides were imaged using a ZOE fluorescence microscope (Bio-Rad, CA, USA).

#### Flow cytometry

FITC-human DCpep was incubated with 10^6^ Mo-DCs for 30 min at 4 °C. After three final washes with PBS, the cells were subjected to flow cytometry (BD Biosciences, San Jose, CA, USA). The experiment was repeated thrice.

### Determination of the capture ability of porcine DCs to recombinant *Lactobacillus* with scanning electron microscope and flow cytometry

#### Scanning electron microscope

Porcine Mo-DCs cultured for 6 d and recombinant *Lactobacillus* were incubated on coverslips for 10, 30, 60, and 120 min. The cells were washed thrice with PBS to remove excess recombinant *Lactobacillus* that did not adhere. Mo-DCs were then fixed with 2% glutaraldehyde at 4 °C and washed thrice with PBS. The samples were freeze-dried after dehydration and dealcoholization, and photographed and analyzed using an SU820 SEM (Hitachi, Tokyo, Japan).

#### Flow cytometry

Carboxyfluorescein succinimidyl ester (CFSE)-labeled (AbMole, Houston, USA) recombinant *Lactobacillus* was incubated with 10^6^ Mo-DCs at 37 °C for 30, 60, and 120 min and washed thrice with PBS; the cells were then subjected to flow cytometry.

### Analysis of marker genes and cytokine expression by Mo-DCs assessed by relative qRT-PCR

Mo-DCs (10^6^/mL) incubated with recombinant *Lactobacillus* at 37 °C for 6, 12, and 24 h were determined by Quantitative real-time RT-PCR (qPCR) using a CFX96™ Real-Time PCR Detection System (Bio-Rad, USA). qPCR was used to quantify the messenger RNA (mRNA) of CD40, CD80, CD86, TLR-2, TLR-4, TLR-6, TLR-9, IL-4, IFN-γ, IL-12, IL-10, and IL-17 in the total RNA, isolated from non-stimulated Mo-DCs and Mo-DCs incubated with recombinant *Lactobacillus* using an RNA Extraction kit according to the manufacturer's instructions (Omega Bio-Tek, Norcross, GA, USA). Reverse-transcription of the total RNA was performed using the PrimeScript™ RT Reagent kit with gDNA Eraser (Takara, Dalian, China) according to the manufacturer’s instructions. Quantitative real-time PCR was performed using the SYBR™ Green PCR Master Mix (Roche, Shanghai, China). The specific primer sequences are listed in **Table 1**. Finally, the 2^-ΔΔct^ method was used to calculate relative gene expression values compared with the β-actin gene control (28).

**Table 1:** Details of the specific primer sequences used for qPCR experiments.

Gene	Primer sequence (5′ -3′)	Accession number
β-actin	F-GGTGGGTATGGGTCAGAAAG R-TCCATGTCGTCCCAGTTGGT	AF054837
CD40	F-CGTGCGGGGACTAACAAGA R-CCAACAGGACGGCAAACA	AF248545
CD80	F-GAGTCCGAATATACTGGCAAAAGG R-AGGTGCGGTTCTCATACTTGG	AF455811
CD86	F-GTGTGGGATGGTGTCCTTTGT R-TTTGTTCACTCGCCTTCCTGT	NM_214222.1
TLR-2	F-ACCATTCCCCAGCGTTTCT R-GAGTCAGCAAGTCACCCTTTATGTT	NM_213761
TLR-4	F-ACCAGACTTTCTTGCAGTGGGTCA R-AATGACGGCCTCGCTTATCTGACA	NM_001113039
TLR-6	F-TCCCAGAATAGGATGCAGTGCCTT R-ACTCCTTACATATGGGCAGGGCTT	NM_213760
TLR-9	F- ACCAGGGACAACCACCACTT R- CAGGCAGAGAGGCAAATCC	XM_005669564.3
INF-γ	F-CGAAAAGCTGATTAAAATTCCGGTA R-TCTTAGGTTAGATCTTGGTGACAGA	NM_213948.1
IL-12	F- TGGACCTCAGACCAGAGCAG R- GCAGGAGTGACTGGCTCAGA	U08317
IL-10	F- GGAAGACGTAATGCCGAAGG R- GGCACTCTTCACCTCCTCCA	NM_214041
IL-17	F-CGGCTGGAGAAAGTGATGGT R-CAGAAATGGGGCTGGGTCT	AB102693

### Evaluation of mixed leukocyte reaction in Mo-DCs

MLR was evaluated using an automated ELISA reader (Bio-Tech Instruments, USA) and a CCK-8 cell counting kit (Beyotime, China). PBMC as reaction cells were obtained from piglet peripheral blood by Ficoll gradient centrifugation, resuspended using RPMI 1640 basal culture medium, and washed thrice. ImMo-DCs were co-cultured with LPS or recombinant *Lactobacillus*, treated with mitomycin C (25 µg/mL) at 37 °C for 1h. The cells were counted and resuspended in complete RPMI 1640 medium, at which point the cells were obtained as stimulated cells. In a 96-well plate, lymphocytes were first added to each well, and Mo-DCs were added at ratios of 1:1, 1:10, and 1:100 for stimulated cells: reactor cells, respectively. Negative control wells were set for Mo-DCs and T cells, and blank control wells were set for RPMI 1640 culture solution. Three replicates per well were incubated at 37 °C in a 5% CO_2_ incubator for 72 h. Finally, CCK-8 was added to the 96-well plates and OD450 values were read on an ELISA reader. The stimulation index (SI) was calculated following the formula: SI = (OD _sample well_ – OD _blank well_) / (OD _negative well_ – OD _blank well_).

### Detection of cytokines in Mo-DCs by ELISA

To detect the secretion of cytokines, the levels of IL-10, IL-12, IFN-γ, and IL-17 were detected in cultures of LPS (2 µg/mL) or recombinant *Lactobacillus*-stimulated Mo-DCs for 24 h using ELISA kits, according to the manufacturer’s instructions and calculated from the curves generated using cytokine standards.

### Statistical analysis

Data are presented as the mean ± standard deviation (SD). Data were analyzed using two-way ANOVA with multiple comparison (LSD) tests in SPSS. Different letters (a vs. b, a vs. c, and b vs. c) indicate significant differences (p < 0.01) at the same time point.

## Results

### Cultured Mo-DCs have the typical morphology and molecular phenotype of DCs

**Figure 1:**
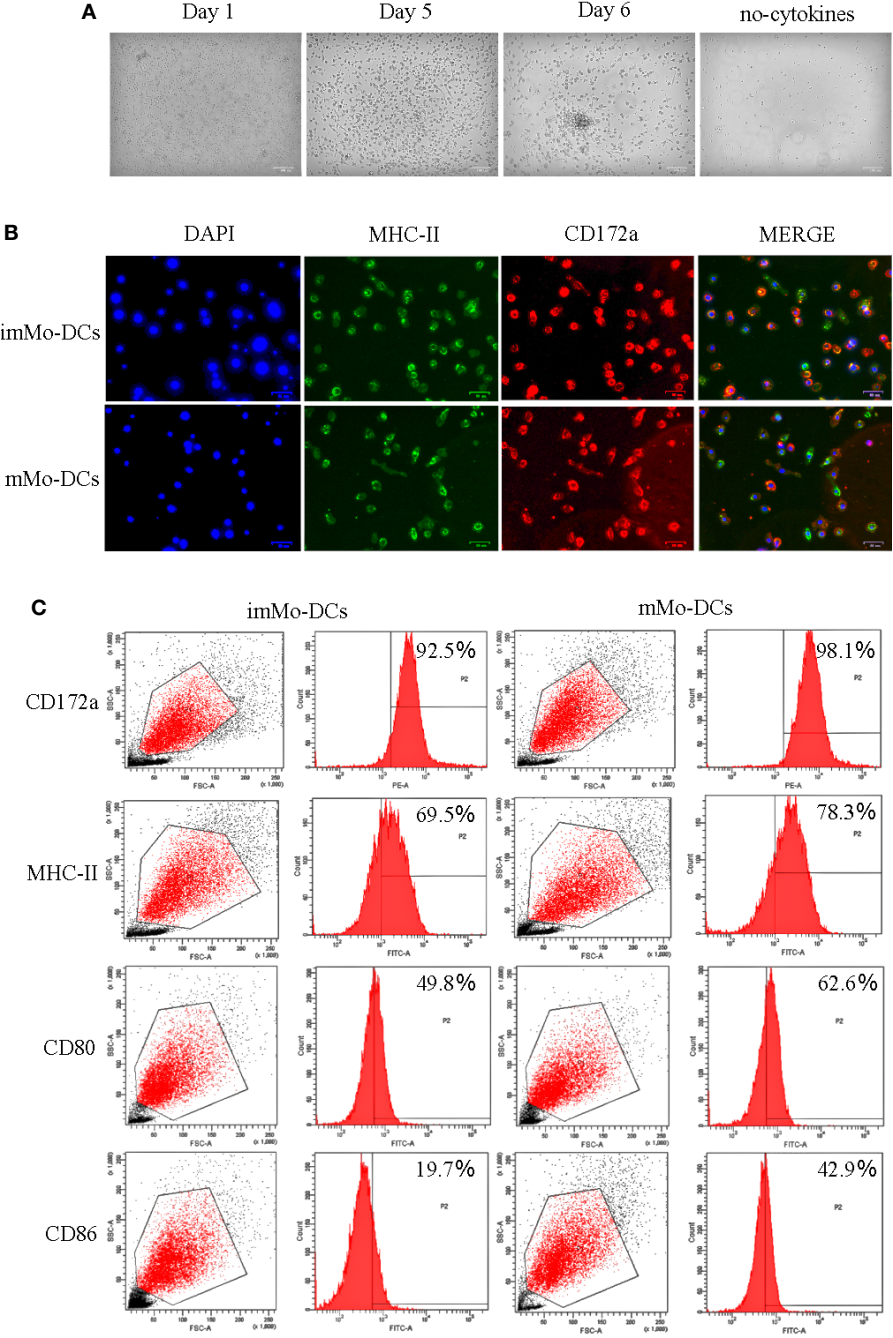
Typical morphology and molecular phenotype of pig monocyte-derived dendritic cells (Mo-DCs). **(A)** Images showing the morphology of immature Mo-DCs (imMo-DCs) (Day 5) and mature Mo-DCs (mMo-DCs) (Day 6, imMo-DCs were treated with LPS for 24 h for maturation) using optical microscopy. Scale bars represent 100 μm. **(B)** ImMo-DCs and mMo-DCs stained with antibodies show the expression of MHC-II (green), CD172a (red), and nuclei stained with DAPI (blue). Scale bars represent 50 μm. **(C)** Representative images of flow cytometry gating for pig Mo-DCs. Surface marker abundance was expressed by % abundance for CD172a, MHC-II, CD80, and CD86 positive populations. Changes to the brightness, contrast, or color balance were applied to every pixel in the image by microscopy.

To obtain Mo-DCs, adherent PBMCs were induced in culture medium containing cytokines for 5 d, followed by LPS stimulation for 1 d. Observation of cell morphology at different time periods under a microscope showed that on Day 1, the cells were round, small, and mostly singular, with smooth surfaces without projections; on Day 5, the cells were semi-suspended or suspended, with a round appearance with dendritic processes; and on Day 6, the cells stimulated by LPS were larger and irregularly shaped, occurred as single cells or clusters, and formed long pseudopodia, all of which are characteristic of DC morphology (**Figure 1A**). To further identify the typical molecular phenotype of Mo-DCs, the expression of surface marker molecules in DCs was detected using fluorescence microscopy and flow cytometry. As expected, both imMo-DCs and mMo-DCs expressed CD172a, MHC-II, CD80, and CD86, and the expression levels of CD80 and CD86 in mMo-DCs were significantly higher than those in imMo-DCs (**Figures 1B, C**). These results indicate that Mo-DCs can be obtained using the above method.

### Human DCpep can target and bind to porcine DCs

**Figure 2:**
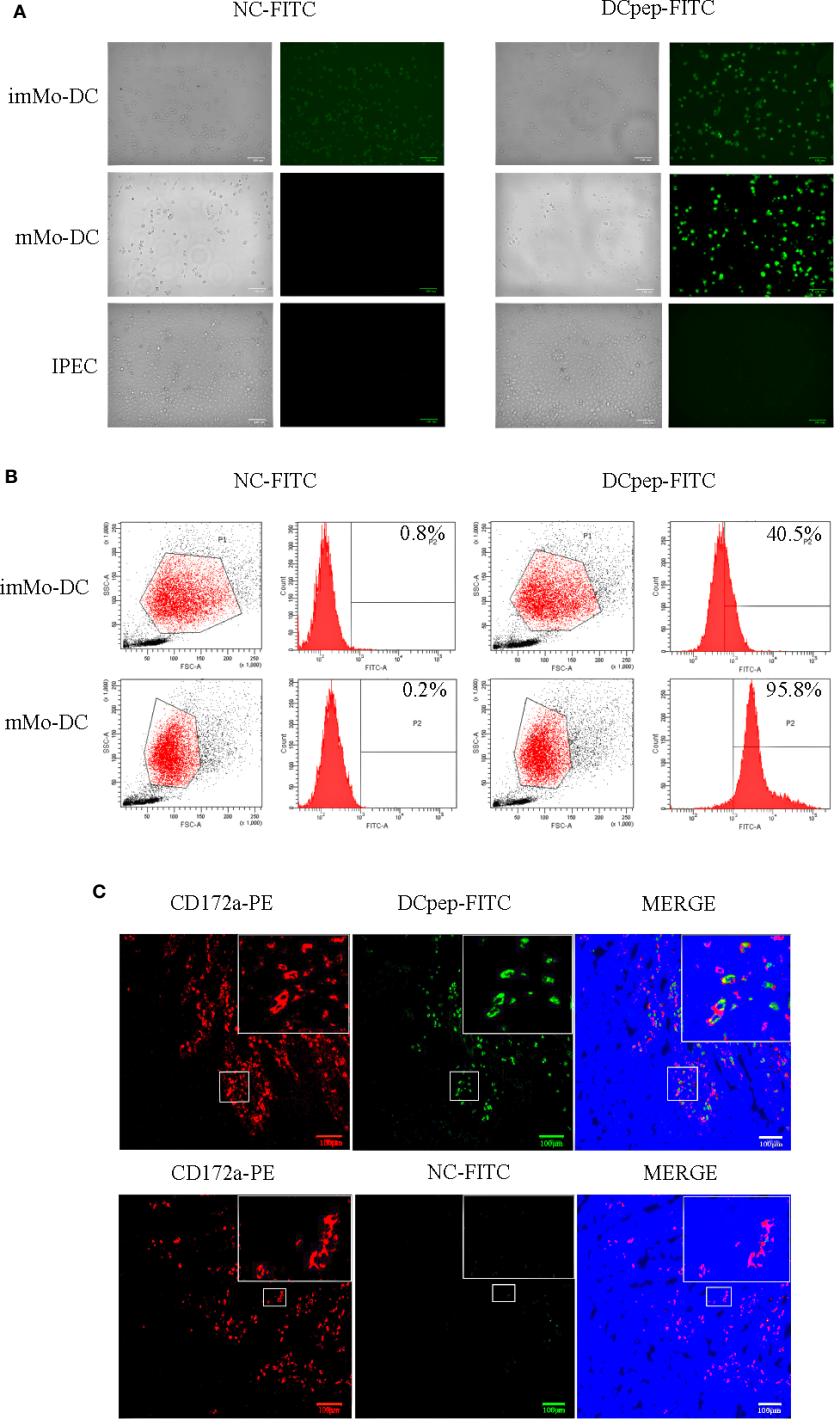
The ability of FITC-labeled human DCpep to bind to pig Mo-DCs was analyzed using fluorescence microscopy and flow cytometry. Fluorescence **(A)** and flow cytometry **(B)** analyses of human DCpep binding to pig Mo-DCs. The human DCpep binding to DCs of the small intestine of healthy piglets was analyzed using fluorescence microscopy **(C)**. The pig DCs expressing CD172a are shown in red. FITC-labeled peptides are shown in green. The nuclei stained with DAPI are shown in blue. Scale bars represent 100 μm. Changes to brightness, contrast, or color balance were applied to every pixel in the image by microscopy.

To verify whether human DCpep could target porcine DCs, DCpep-FITC and NC-FITC were incubated with imMo-DCs and mMo-DCs. The binding of human DCpep to Mo-DCs was detected using fluorescence microscopy and flow cytometry. The results showed that DCpep-FITC can bind to imMo-DCs and mMo-DCs, but not to IPEC (**Figures 2A, B**). To detect the binding ability of human DCpep to DCs in tissue, frozen sections were prepared from the small intestine of healthy piglets. DCpep-FITC was incubated with the frozen sections, and DCs were labeled with anti-pig CD172a-PE (29). The results of fluorescence microscopy (**Figure 2C**) showed that human DCpep binds to DCs in the small intestine of piglets but not to other cells in the tissue. In summary, human DCpep binds to porcine DCs.

### Human DCpep improves the efficiency of antigen capture of porcine DCs

**Figure 3:**
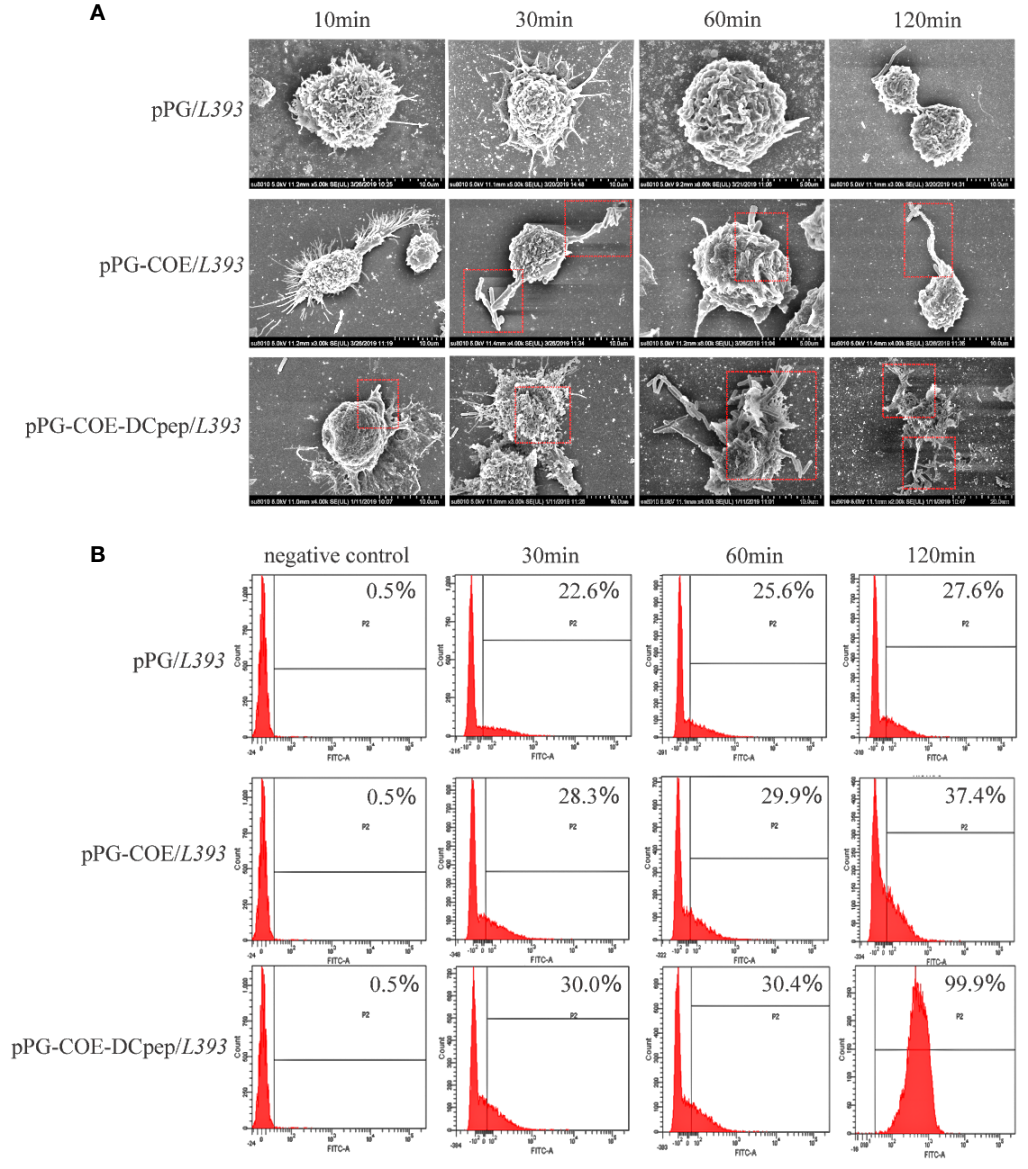
The ability of Mo-DCs to recognize and capture recombinant *Lactobacillus* was evaluated using scanning electron microscopy and flow cytometry. **(A)** Scanning electron microscopy images showing the morphology of Mo-DCs capturing recombinant *Lactobacillu*s at 10, 30, 60, 120 min. **(B)** Mo-DCs capturing recombinant *Lactobacillus* detected by flow cytometry. Mo-DC surface recombinant *Lactobacillus* abundance was expressed by % abundance for pPG/*L393*, pPG-COE/*L393*, and pPG-COE-DCpep/*L393* positive populations.

To determine the effect of human DCpep on antigen recognition and capture efficiency of porcine DCs, recombinant *Lactobacillus* was incubated with Mo-DCs. The ability of Mo-DCs to recognize and phagocytize recombinant *Lactobacillus* was observed using scanning electron microscopy. **Figure 3A** shows that Mo-DCs recognize and phagocytize pPG-COE/*L393* and pPG-COE-DCpep/*L393*; the efficiency of Mo-DCs to capture pPG-COE-DCpep/*L393* was significantly higher than that of pPG-COE/*L393*. For a more accurate quantitative analysis, the amount of Mo-DCs that successfully captured recombinant *Lactobacillus* was detected by flow cytometry; the results showed that the number of Mo-DCs that captured pPG-COE-DCpep/*L393* was significantly higher than that for pPG-COE/*L393*. At 120 min, almost all Mo-DCs captured pPG-COE-DCpep/*L393* (**Figure 3B**). These results show that human DCpep can improve the efficiency of antigen recognition and capture of porcine DCs.

### Fusion of human DCpep with antigens increases the expression levels of markers of porcine DCs

**Figure 4:**
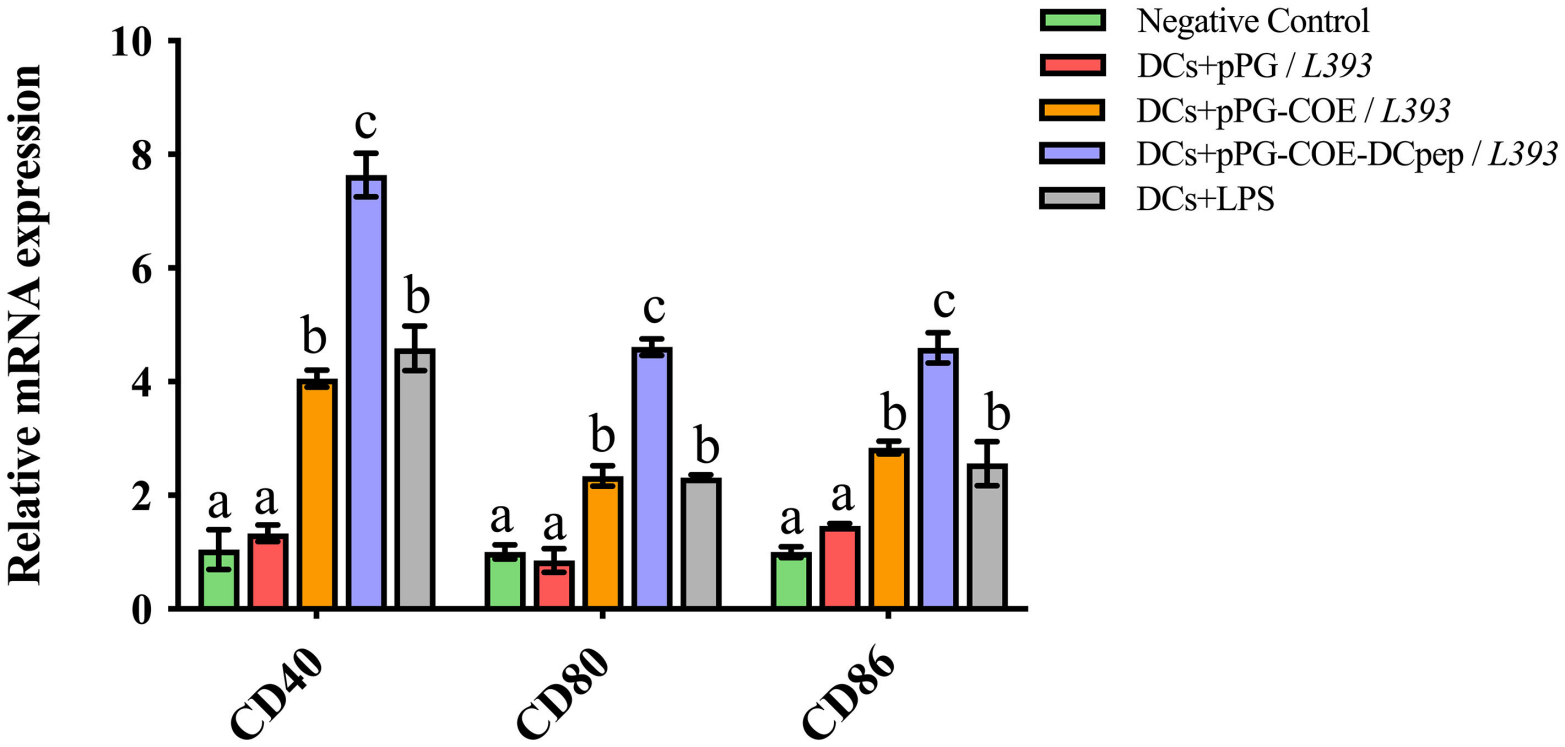
Marker expression of Mo-DCs (10^6^ cells/ mL) stimulated by pPG/*L393*, pPG-COE/*L393*, pPG-COE-DCpep/*L393*, and LPS (2 µg/mL) assessed by relative qRT-PCR, recombinant *Lactobacillus*-incubated DCs at a ratio of 1:10 (DCs:recombinant *Lactobacillus*). Different letters (a vs. b, a vs. c, b vs. c) indicate significant differences (p < 0.01) at the same time point.

We tested the marker expression of Mo-DCs stimulated by recombinant *Lactobacillus* expressing human DCpep or the control peptide using real-time RT-PCR. Upregulation of Mo-DC marker genes, such as CD40, CD80, and CD86, indicates DC maturation. The results of real-time RT-PCR (**Figure 4**) showed that CD40, CD80, and CD86 expression in pPG-COE-DCpep/*L393*-stimulated Mo-DCs was significantly higher than that in the pPG-COE/*L393* group at 12 h (p <0.01). These results indicate that fusion of human DCpep with antigens can promote DC maturation.

### Fusion of human DCpep with antigens improves the antigen presentation efficiency of porcine DCs

**Figure 5:**
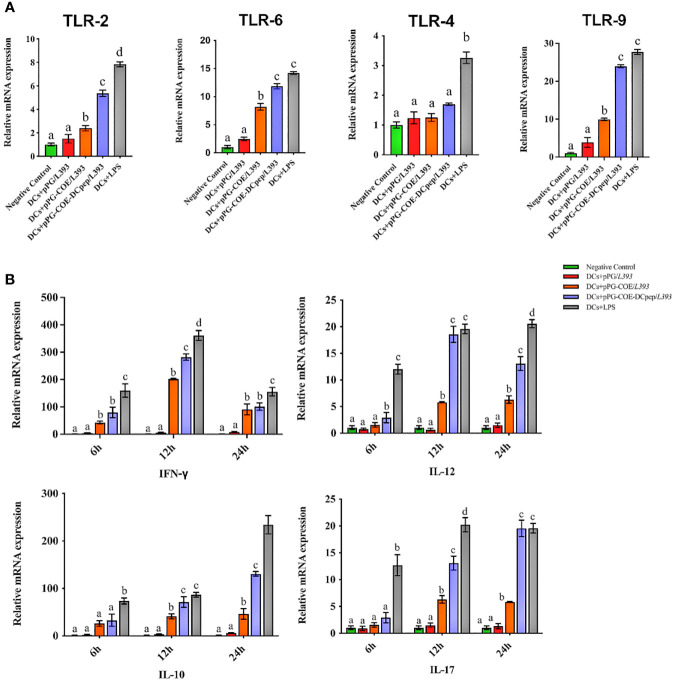
Analysis of toll-like receptor **(A)** and cytokine **(B)** mRNA levels in Mo-DCs (10^6^ cells/ mL) in response to pPG/*L393*, pPG-COE/*L393*, pPG-COE-DCpep/*L393*, and LPS (2 µg/mL) stimulation; recombinant *Lactobacillus*-incubated DCs at a ratio of 1:10 (DCs: recombinant *Lactobacillus*). Mo-DCs were stimulated by recombinant *Lactobacillus* and LPS for 6, 12, or 24 h. Unstimulated Mo-DCs were used as a control. Different letters (a vs. b, a vs. c, b vs. c) indicate significant differences (p < 0.01) at the same time point.

Toll-like receptors (TLRs) that are expressed and cytokines that are released by DCs are important indicators of immune responses. The mRNA levels of TLRs and cytokines expressed by recombinant *Lactobacillus-*stimulated Mo-DCs were detected at 6 h using real-time RT-PCR. TLRs on the surface of DCs play a central role in receiving external stimulation signals and inducing an immune response. The results of real-time RT-PCR (**Figure 5A**) showed that TLR-2, TLR-6, and TLR-9 expression in Mo-DCs was higher in the pPG-COE-DCpep/*L393* group than in the other groups compared to the pPG-COE/*L393* group (p <0.01).

Cytokines released by DCs are involved in immune responses and have a critical effect on the differentiation of naïve T cells into Th1 and Th2 cells. The results of real-time RT-PCR (**Figure 5B**) showed that the mRNA levels of Th1-associated cytokines (IFN-γ and IL-12) and Th17-associated cytokines (IL-17) were significantly higher in the pPG-COE-DCpep/*L393* groups than in the pPG-COE /*L393* groups at 12 h. IFN-γ has antiviral activity and can upregulate the expression of MHC antigens. IL-12 plays an important role in the activities of natural killer cells and T lymphocytes. In addition, changes in the expression of anti-inflammatory cytokines (IL-10) in Mo-DCs with pPG-COE-DCpep/*L393* gradually increased over time. Based on these results, we conclude that human DCpep can direct more antigens to porcine DCs.

### Fusion of human DCpep with antigens promotes porcine DC-mediated T cell differentiation into Th1 cells

**Figure 6:**
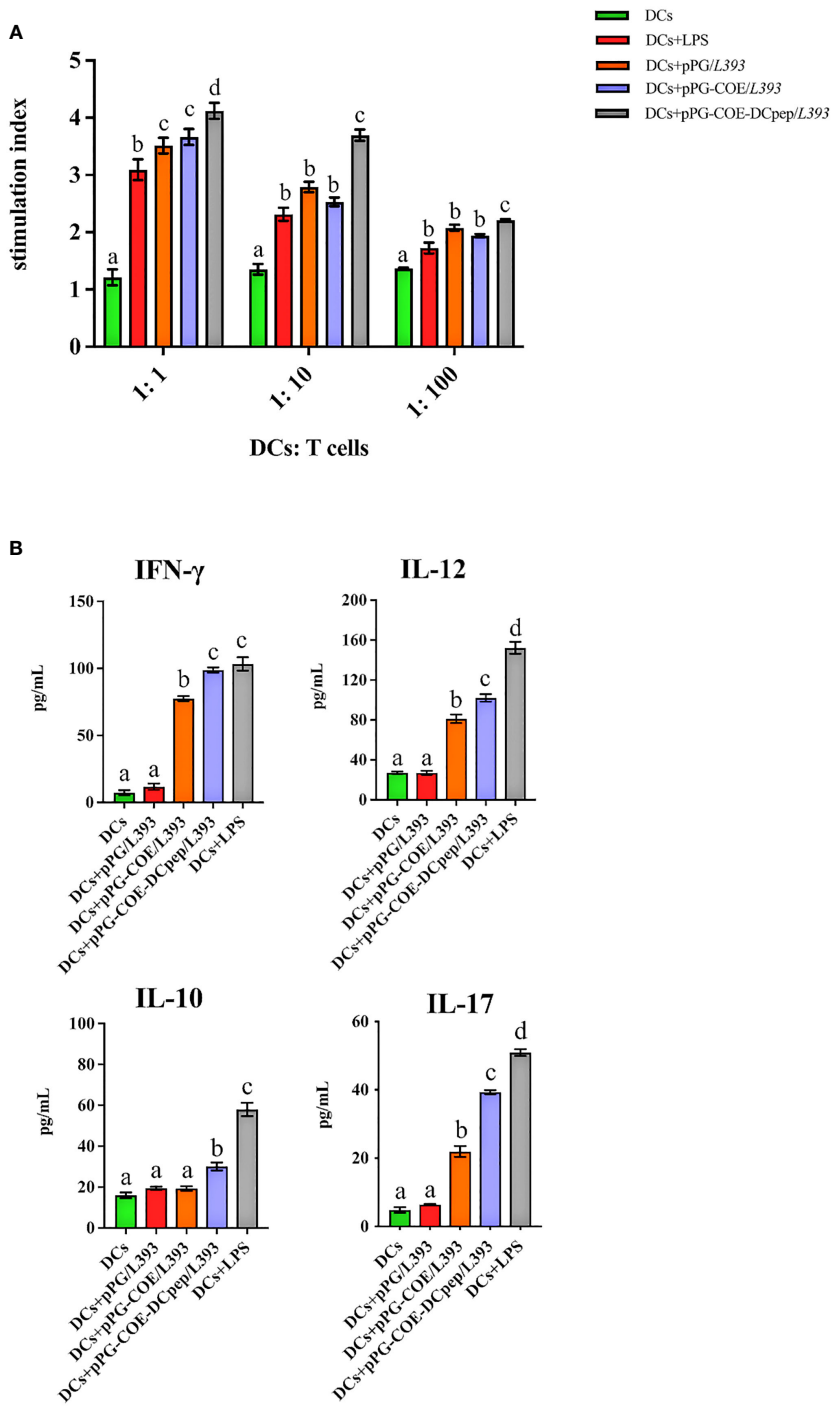
Analysis of Mo-DCs treated with pPG/*L393*, pPG-COE/*L393*, pPG-COE-DCpep/*L393*, and LPS skew T cells toward different effector T cell profiles. **(A)** Mo-DCs treated with recombinant *Lactobacillus* and LPS stimulate the proliferation of T lymphocytes in mixed lymphocyte reaction (MLR). Responder cells were added at ratios of 1:1, 1:10, or 1:100 and co-cultured with the stimulated cells for 72 h. Proliferation is expressed as the Stimulation Index (SI) calculated using the formula: SI = (OD_sample_ − OD_stimulator cells only_) / (OD_responder cells only_ − OD_blank control_). All experiments were performed at minimum in triplicate. Data are presented as mean ± SEM (n = 6 per group). **(B)** MoDCs were stimulated with recombinant *Lactobacillus* and LPS for 12 h, and then co-cultured with allogenic T cells at a ratio of 1:10. After 72 h, culture supernatants were collected and analyzed for cytokines by ELISA. Different letters (a vs. b, a vs. c, b vs. c) indicate significant differences (p < 0.01) at the same time point.

We further assessed whether the fusion of human DCpep with antigens affects the type of mediated immune response. We prepared a single-cell suspension of lymphocytes from piglets and performed cell proliferation tests. The results showed that pPG-COE-DCpep/*L393* resulted in significantly higher levels of T cell proliferative responses than pPG-COE /*L393* (P < 0.05) (**Figure 6A**). This result implies that the fusion of human DCpep with antigens enhanced the ability of porcine DCs to activate T-cell proliferation.

To further determine the type of T-cell differentiation mediated by Mo-DCs stimulated by recombinant *Lactobacillus*, we measured the levels of IFN-γ, IL-12, IL-10, and IL-17 using ELISA kits. **Figure 6B** shows that recombinant *Lactobacillus* pPG-COE-DCpep/*L393* induced the secretion of a significant number of cytokines (IFN-γ, IL-12, IL-10, and IL-17) by DCs and T cells. In addition, the secretion of IFN-γ and IL-12 was higher than that of IL-10 and IL-17 by Mo-DCs induced by recombinant *Lactobacillus.* From the above results, it can be concluded that, with the participation of human DCpep, recombinant *Lactobacillus* stimulates Mo-DCs to mediate the differentiation of more T cells into Th1 cells.

## Discussion

In recent years, DC-targeting strategies have become a research hotspot, reflecting the important role of DCs in inducing and regulating the immune response. Significant progress has been made in the development of human DC-targeted vaccines. This strategy can increase the number of antigens targeted by DCs. As a result, the vaccine dose can be appropriately reduced and a strong and rapid immune response can be induced simultaneously (30–32). Since oral vaccines for pigs have the advantage of not only reducing production costs, but also reducing animal stress, DC-targeting vaccines have become a desirable choice (33). Our laboratory has previously studied the effects of recombinant *Lactobacillus* expressing target antigens; human DCpep can induce stronger mucosal and humoral immune responses in pigs than recombinant *Lactobacillus* without DCpep (34, 35); however, the immune mechanism is not clear. Hence, we explored the mechanism by which human DCpep enhances the immune effects of antigens on porcine DCs.

Researchers found that human DCpep could be targeted to rhesus monkey and chimpanzee bone marrow derved dendritic cells, but not to T cells, B cells, or monocytes (26). Pigs, as mammals very similar to humans, are anatomically and genetically close to humans, and the mechanisms of dendritic cell-induced immune reactions should be similar in mechanism. (36). In recent years, some studies have attempted to apply human DCpep to porcine vaccines to enhance the immune response through oral administration of recombinant *Lactobacillus* expressing human DCpep (24, 37, 38). Our previous study showed that recombinant *Lactobacillus* expressing human DCpep induced significantly enhanced immune responses than recombinant *Lactobacillus* without DCpep (34). The present results revealed that human DCpep could targeted to porcine DC. This suggest porcine and human DCs are, to some degree, homologous and have the same ligands for human DCpep. It has been shown that the ligands of DCpep are involved in the endocytic pathway of cells, allowing more efficient transport of immunogenic substances into the cells without impairing DC function (19).

DC-targeting strategies for delivering protective antigens to DCs and stimulating stronger and longer lasting immune responses have received increasing attention (23, 39). DCs, as attractive targets for vaccine design, have many features, such as the ability to induce T cell activation and differentiation through the presentation of antigenic peptides by MHC molecules to initiate adaptive responses (40). In this study, we observed, using scanning electron microscopy, that recombinant *Lactobacillus* pPG-COE-DCpep/*L393* effectively stimulated Mo-DCs to generate synapses and capture more recombinant *Lactobacillus*. As DCs mature, they upregulate mechanisms of antigen presentation, including MHC-II, costimulatory molecules, and pro-inflammatory cytokines, and subsequently migrate to the T-cell zone of secondary lymphoid tissue where they stimulate antigen-specific T cells. (41). The recombinant *Lactobacillus* and pPG-COE-DCpep/*L393* may act by promoting Mo-DC maturation, because they significantly upregulate the expression of MHC-II, CD80, and CD86 on imMo-DCs.

A previous study showed that TLRs bridge the gap between non-specific and specific immunity and play an irreplaceable role in the activation of DCs using recombinant *Lactobacillus*. TLR-9 recognizes bacterial CpG-DNA and activates the immunostimulatory properties of B cells and APCs (21). In this study, we evaluated the expression of TLRs (TLR-2, TLR-4, TLR-6, and TLR-9) in Mo-DCs stimulated with recombinant *Lactobacillus* by real-time RT-PCR analysis; we found that recombinant *Lactobacillus* pPG-COE-DCpep/*L393* increased the expression of TLR-2, TLR-6, and TLR-9 in Mo-DCs to stimulate the host mucosal immune system. However, there was no change in TLR-4 expression. This may be due to the fact that TLR2 forms a heterodimer with TLR1 or TLR6 and recognizes a wide variety of microbial ligands, while TLR4 is the main receptor that mediates the endotoxin/LPS response, so its expression is not affected by gram-positive bacteria (42).

The cytokine response induced by recombinant *Lactobacillus-*stimulated Mo-DCs is another critical process that results in antigen presentation to activate T cells. IFN-γ, a cytokine produced by natural killer cells and T lymphocytes, can enhance the phagocytic activity to efficiently kill pathogens. Reportedly, Th1 cells mainly secrete IFN-γ and TNF-β. IFN-γ activates macrophages and inhibits the proliferation of Th2 lymphocytes. It also stimulates B cells to produce receptors that enhance the microbial attachment to phagocytes (43). IL-12 is the most important cytokine in Th1 differentiation, promoting the survival and growth of Th1 cells and maintenance of sufficient numbers of memory/effector Th1 cells. In addition, IL-12 can suppress Th2 cell formation (44). IL-10 is a major Th2-type cytokine that promotes Th2 cell proliferation and inhibits Th1 cell proliferation, while supporting the activation of B cells and playing a role in humoral immunity (45). In this study, we evaluated the expression of cytokines (IL-12, IFN-γ, and IL-10) in Mo-DCs stimulated with recombinant *Lactobacillus* by real-time RT-PCR analysis and found that recombinant *Lactobacillus* expressing DCpep increased the expression of IL-12, IFN-γ, and IL-10 in Mo-DCs. The results showed that recombinant *Lactobacillus* pPG-COE-DCpep/*L393* can significantly promote the maturation of Mo-DCs and secretion of cytokines. The results also suggest that recombinant *Lactobacillus* expressing DCpep triggers a stronger immune response in Th1 and Th2 type cells.

Several studies have shown that antigens conjugated with DC-targeting antibodies can more rapidly activate and promote the proliferation of CD4^+^T cells (46–48). In line with previous reports, our results showed that recombinant *Lactobacillus* pPG-COE-DCpep/*L393* significantly induced Mo-DCs to stimulate T-cell proliferation at a DC/T ratio of 1:1. From these results, we observed that the greater the number of Mo-DCs, the stronger is the ability to activate T cell proliferation. Thus, there is a minor but significant difference between the recombinant *Lactobacillus* pPG-COE-DCpep/*L393* stimulated Mo-DCs at a 1:1 DC/T ratio, but not at the other ratios. There are different subpopulations of Th cells, including Th1, Th2, Th17, and regulatory T cells, each activated by a specific set of cytokines and transcription factors, and characterized by the cytokines they secrete and the effector functions (49, 50). In this study, the levels of secreted IL-12 and IFN-γ by the recombinant *Lactobacillus* pPG-COE-DCpep/*L393* stimulated Mo-DCs were higher than those of IL-10 and IL-17, suggesting that DCpep is beneficial to recombinant *Lactobacillus* to stimulate DCs to mediate the cellular immune response and the differentiation of T cells into Th1 type cells.

In conclusion, our results demonstrate that human-derived DCpep could not only be targeted to porcine DCs but also be combined with porcine monocytes and lymphocytes; these findings suggest that human-derived DCpep can significantly promote antigen presentation in porcine DCs and improve the efficiency of recombinant *Lactobacillus* to deliver antigens to porcine DC. Furthermore, human-derived DCpep can activate porcine DCs and play a key role in the cellular immune response. In summary, our study provides a robust theoretical basis for the development of porcine DC-targeted vaccines.

## Data availability statement

The original contributions presented in the study are included in the article/supplementary material. Further inquiries can be directed to the corresponding authors.

## Ethics statement

This study was reviewed and approved by the Animal Experiment Ethics Committee of Northeast Agricultural University, China.

## Author contributions

YL and LW conceived and designed the study. TX, HY, YG, TG, LX, YJ, WC, HZ, and XQ performed the experiments. XW, JL, ZS, and LT interpreted and analyzed the data. TX wrote the manuscript. All authors contributed to the article and approved the submitted version.

## Funding

This work was supported by the National Natural Science Foundation of China (31972718).

## Conflict of interest

The research was conducted in the absence of any commercial or financial relationships that could be construed as a potential conflict of interest.

## Publisher’s note

All claims expressed in this article are solely those of the authors and do not necessarily represent those of their affiliated organizations, or those of the publisher, the editors and the reviewers. Any product that may be evaluated in this article, or claim that may be made by its manufacturer, is not guaranteed or endorsed by the publisher.
